# Carotid endarterectomy versus carotid angioplasty for stroke prevention: a systematic review and meta-analysis

**DOI:** 10.1186/s13019-016-0532-x

**Published:** 2016-09-08

**Authors:** Zengyan Diao, Guoyong Jia, Wei Wu, Cuilan Wang

**Affiliations:** Department of Neurology, Qilu Hospital of Shandong University, Jinan, 250012 Shandong Province China

**Keywords:** Carotid angioplasty, Carotid endarterectomy, Meta-analysis

## Abstract

**Background:**

This meta-analysis aimed to evaluate the efficacy of carotid endarterectomy (CE) compared with carotid angioplasty (CA) in preventing stroke. Whether the use of CE is more efficient in preventing stroke than CA is a matter of debate.

**Methods:**

Data were gathered from randomized controlled trials to evaluate the effect of CE compared with CA on the risk of stroke. Electronic searches in PubMed, Embase, and the Cochrane Library were performed to identify studies till November 2014. Only randomized controlled trials performed on patients who received either CE or CA for stroke prevention were included.

**Results:**

Nine relevant trials (*n* = 7163) that met the inclusion criteria were identified. In a pooled analysis, CE resulted in 35 % reduction in relative risk (RR) for short-term stroke [RR, 0.65; 95 % confidence interval (CI): 0.47–0.89; *P* = 0.007)] and 22 % reduction in RR for long-term stroke (RR, 0.78; 95 % CI: 0.66–0.93; *P* = 0.006) relative to CA. However, CE also increased the risk of 30-day myocardial infarction by 114 % compared with CA (RR, 2.14; 95 % CI: 1.30–3.53; *P* = 0.003). Sensitivity analyses suggested that CE might influence the risk of 30-day major vascular events and 1-year major vascular events compared with CA.

**Conclusions:**

CE could reduce the risk of stroke (whether short term or long term), but resulted in a relative increase in the risk of myocardial infarction. This study might guide appropriate judgments about treatment approach. It also provided evidence to justify general guidelines for patients with carotid artery stenosis.

## Background

Cerebrovascular disease, either ischemic or hemorrhagic stroke, is the leading cause of premature mortality and morbidity worldwide for both men and women [1–3]. Asian countries have a higher incidence of stroke compared with Western countries [4]. Over the past few years, many studies have shown a strong correlation between carotid artery stenosis and stroke [5, 6]. It has been suggested that carotid artery stenosis should be corrected as a therapeutic approach to prevent stroke events. However, the use of carotid endarterectomy (CE) compared with carotid angioplasty (CA) for preventing stroke has not been shown consistently to be beneficial.

CE was recommended as the standard therapy, which could reduce the risk of stroke in patients with carotid artery stenosis [7]. However, in many cases, a high residual risk of stroke persists after CE. Hence, it is necessary to explore additional effective preventive therapies [8]. Recently, endovascular treatments [9] (CA with or without stenting) have been increasingly used as an alternative to CE. However, whether endovascular treatments are more effective than surgery in patients with carotid artery stenosis remains unclear. This led to uncertainty over the presence and magnitude of any protective effects of endovascular treatments and surgery on stroke, and also difficulties in interpretation of the results. Therefore, this systematic review and meta-analysis was conducted to evaluate the possible effect of CE compared with CA on stroke in patients with carotid artery stenosis.

## Methods

### Data sources, search strategy, and selection criteria

This review was conducted and reported according to the Preferred Reporting Items for Systematic Reviews and Meta-Analysis Statement [10] issued in 2009. Data were gathered from randomized controlled trials to evaluate the effect of CE compared with CA on the risk of stroke. Trials comparing CE with CA were included, excluding any studies with a sample size less than 50, to alleviate systematic error and resulting bias, hence ensuring the reliability of the conclusion.

The English literature was systematically searched to identify all relevant randomized, controlled trials regardless of publication status (published, in press, and in progress). Relevant trials were identified using the following procedures:

1. Electronic searches: The PubMed, Embase, and the Cochrane Central Register of Controlled Trials were searched for randomized controlled trials of CE compared with CA, using “endarterectomy,”, “angioplasty,” “stenting,” stenosis,” “carotid,” “human,” “English,”, and “randomized controlled trials” as search terms. All reference lists from reports on nonrandomized controlled trials were searched manually for additional eligible studies.
2. Other sources: Authors were contacted to obtain any possible additional published or unpublished data, and the site http://www.ClinicalTrials.gov was searched for ongoing randomized controlled trials that had been registered as completed but not yet published, using the aforementioned terms. Medical subject headings, methods, patient populations, interventions, and outcome variables of these studies were used to identify relevant trials.

The literature search, data extraction, and quality assessment were undertaken independently by two authors (CZ and FLC) using a standardized approach, and any discrepancy was settled by group discussion. Studies were eligible for inclusion if: (1) the study was a randomized controlled trial; (2) sample size was more than 50; (3) the number of events for stroke that occurred during the study was more than 10; (4) the trials assessed the effects of CE compared with CA; and (5) patients had carotid artery stenosis.

### Data collection and quality assessment

All data from eligible trials were independently abstracted in duplicate by two independent investigators (CZ and FLC) using the standard protocol, and reviewed by a third investigator (AJH). Any discrepancy was resolved by group discussion. Data were extracted from the included trials were as follows: name of first author or study group, publication year, number of patients, percentage of males, mean age, history of disease, intervention, control, duration of follow-up, and primary outcome (the number of incident cases for each treatment group). One author (AJH) entered the data into computer, and the primary author (YHZ) checked it. The study quality was assessed using the Jadad score [11], which was based on the following five subscales: randomization (1 or 0), concealment of the treatment allocation (1 or 0), blinding (1 or 0), completeness of follow-up (1 or 0), and the use of intention-to-treat analysis (1 or 0). A “score system” (ranging from 0 to 5) was developed for assessment. In this meta-analysis, a study with a score of 4 or more was considered to be of high quality.

### Statistical analysis

The results of each randomized controlled trial was compiled as dichotomous frequency data. Individual study relative risks (RRs) and 95 % confidence intervals (CIs) were calculated from event numbers extracted from each trial before data pooling. The overall RR and 95 % CIs of stroke incidence, major vascular events, myocardial infarction, and any possible adverse events were also calculated. Both fixed-effects and random-effects models were used to assess the pooled RR for CE compared with CA. Although both models yielded similar findings, the results from the random-effects model assumed that the underlying effect varied among included trials [12, 13]. The heterogeneity of the treatment effects between studies was investigated visually using scatter plot analysis as well as statistically using the heterogeneity I^2^ statistic [14, 15]. A sensitivity analysis was also performed by removing each individual trial from the meta-analysis. An Egger’s test [16] was used to check for potential publication bias. All the reported *P* values were two-sided, and a *P* value less than 0.05 was regarded as statistically significant for all included studies. All analyses were calculated using software STATA (version 10.0).

## Results

**Fig. 1:**
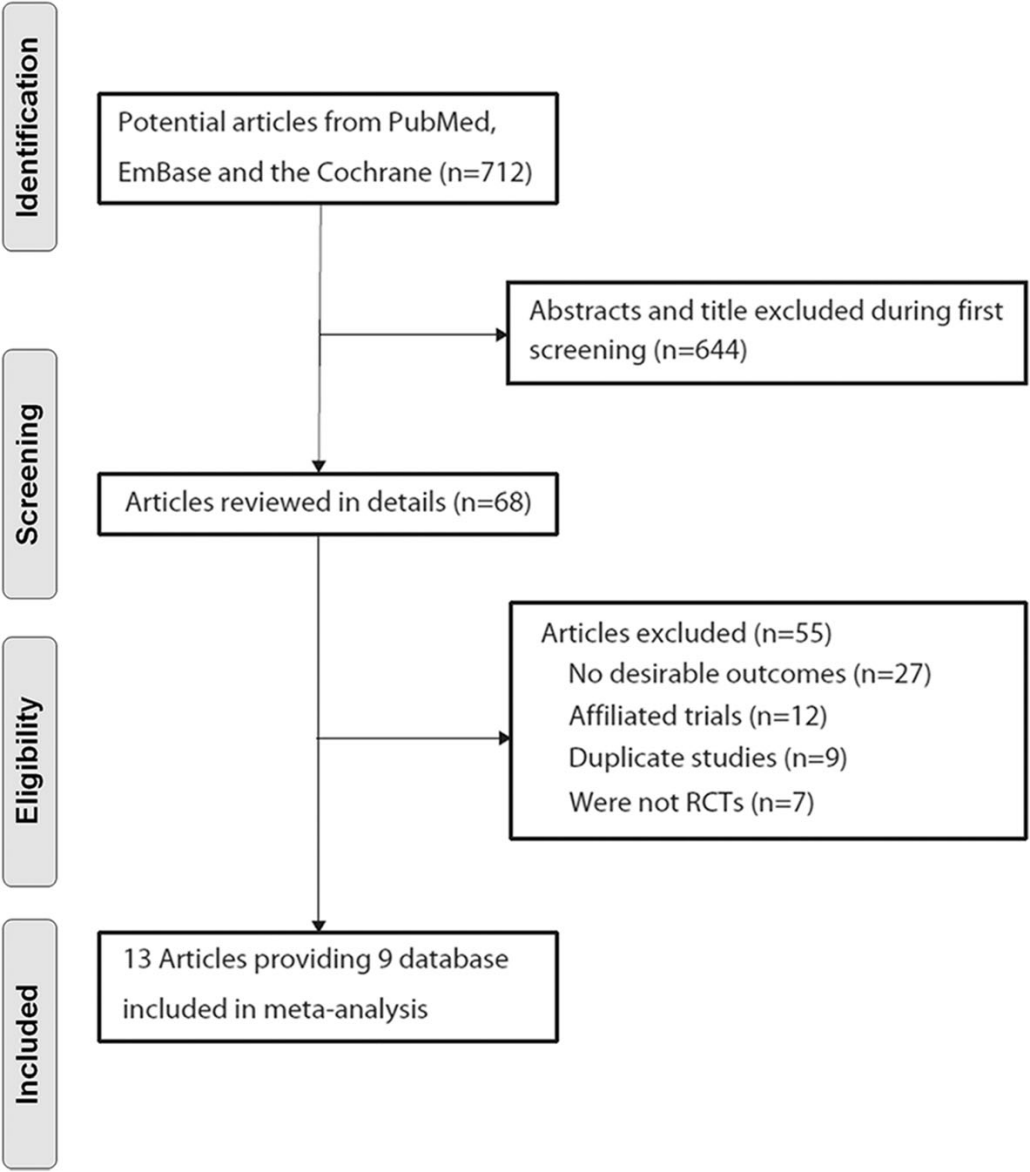
Flow diagram of the literature search and trials selection process

Of the 19 trials retrieved for detailed assessment, 10 were excluded because: they lacked data on stroke, they reported on the same study population, [17] they were of small sample size, or it was a stopped trial. The final analysis included nine randomized controlled trials [18–26] consisting of 7163 patients with carotid artery stenosis (Fig. 1). These trials compared CE with CA, with stroke reported as one of the endpoints. Table 1 summarizes the characteristics of these trials and the important baseline information of the included 7163 patients. Of the nine trials, two were performed in the USA [18, 20], four in European countries [19, 21, 22, 24], one [23] in Germany, Austria, and Switzerland, one [25] in the USA and Canada, and one [26] in Europe, Australia, and Canada. The number of patients ranged from 87 to 2502. The percentage of previous cases with cardiovascular disease ranged from 11.9 to 80.7 %. The duration of follow-up ranged from 0.3 to 5.4 years. The inclusion criteria were restricted to randomized controlled trials with the number of patients more than 50 to ensure that high-quality literature was included in the study. Although the included trials scarcely reported on the key indicators of trial quality, the quality of the included trials was also evaluated according to the predefined criteria using the Jadad score [11]. Overall, five [20–23, 25] of the included trials scored 4, two trials [19, 26] scored 3, and the remaining two trials [18, 24] scored 2.

**Table 1:** Design and characteristic of trials included in the meta-analysis

Source	Publication year	Country	No. of patients	Sex (male, %)	Mean age (year)	History of disease	Previous CVD (%)	Intervention	Control	Follow-up (year)	Jadad score
SAPPHIRE Investigators [18, 19]	2004	US	334	67.0	72.6	Symptomatic carotid artery stenosis of at least 50 % of the luminal diameter or an asymptomatic stenosis of at least 80 %	80.7	Endarterectomy	Carotid artery stenting with an embolic protection device	3	2
MGM Steinbauer [20]	2008	Germany	87	NG	68.5	Symptomatic high-grade internal carotid artery stenosis of at least 70 %	43.7	Endarterectomy	Carotid artery stenting	5.4	3
WH Brooks [21]	2001	US	104	NG	68.0	Symptomatic carotid artery stenosis of at least 70 %	67.3	Endarterectomy	Carotid artery stenting	2	4
EVA-3S Investigators [22, 23]	2006	France	520	75.2	69.7	Symptomatic carotid artery stenosis of at least 60 %	11.9	Endarterectomy	Carotid artery stenting	0.5	4
ICSS Investigators [24]	2010	UK	1710	70.5	70.0	Symptomatic carotid artery stenosis of at least 50 %	18.0	Endarterectomy	Carotid artery stenting	0.3	4
The SPACE Collaborative Group [25, 26]	2006	Germany, Austria, and Switzerland	1183	71.8	67.9	Symptomatic carotid artery stenosis of at least 50 %	22.6	Endarterectomy	Carotid artery stenting	2	4
MJ Alberts [27]	2001	UK	219	NG	NG	Symptomatic carotid artery stenosis of 60–99 %	NA	Endarterectomy	Carotid artery stenting	1	2
The CREST Investigators [28]	2010	US and Canada	2502	65.1	69.0	carotid artery stenosis of at least 50 %	43.7	Endarterectomy	Carotid artery stenting	2.5	4
CAVATAS Investigators [29, 30]	2001	Europe, Australia, and Canada	504	69.5	67.0	carotid artery stenosis	18.0	Endarterectomy	Endovascular treatment	5	3

*CVD* cardiovascular disease, *NA* not available

**Fig. 2:**
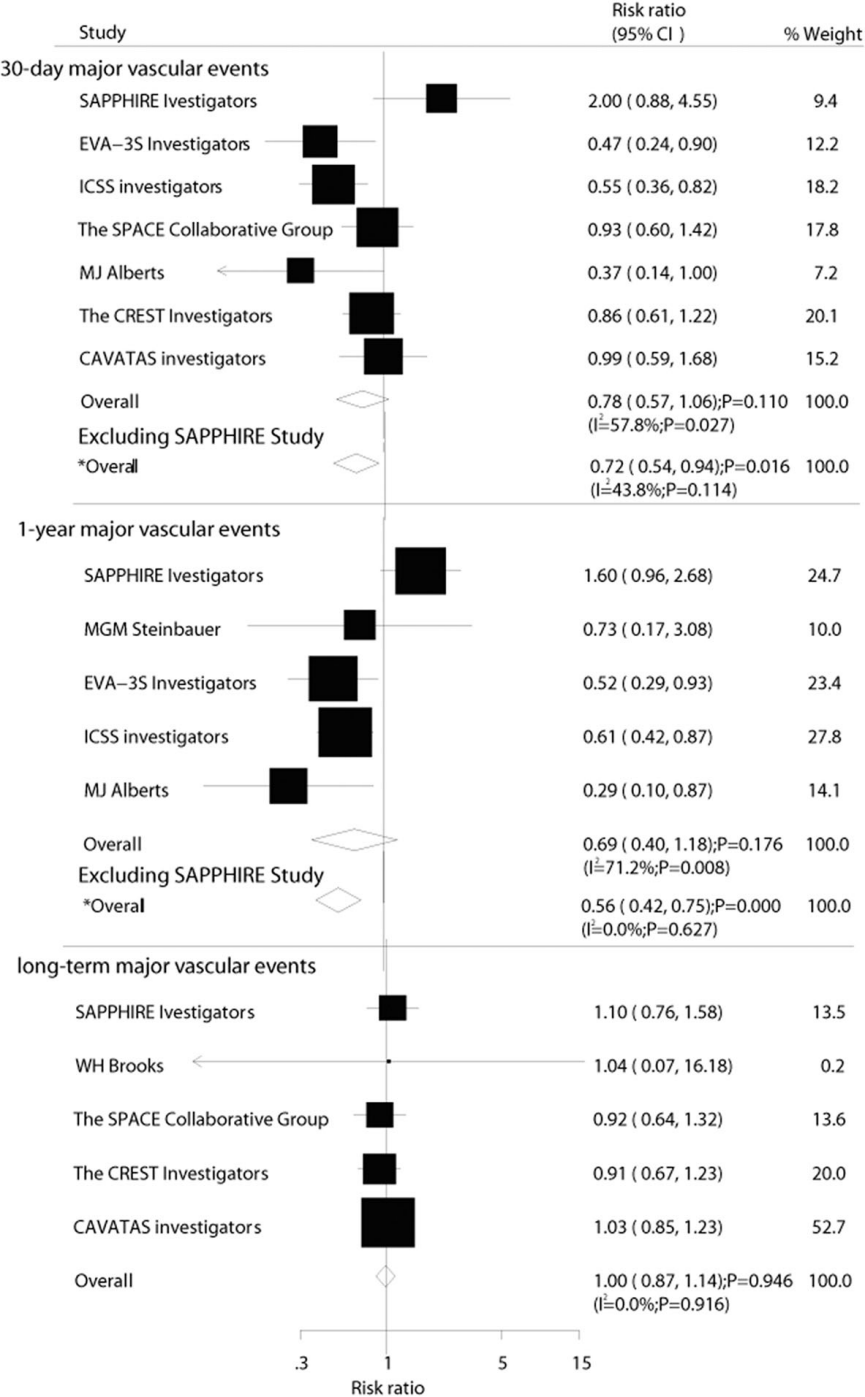
Effect of carotid endarterectomy on the risk of major vascular events compared with carotid angioplasty

Data on the effect of CE on 30-day major vascular events were available from 7 trials, which included 6911 patients and reported 424 major vascular events. Figure 2 shows the effect of CE on 30-day major vascular events compared with CA. The pooled RR showed a 22 % reduction in 30-day major vascular events, but with no evidence showing that CE protected against the risk of vascular events (RR, 0.78; 95 % CI: 0.57–1.06; *P* = 0.11). Some evidence of heterogeneity across the studies included was available. A sensitivity analysis indicated that CE was associated with a reduction in the risk of 30-day major vascular events, which was decreased by 28 % (RR, 0.72; 95 % CI: 0.54–0.94; *P* = 0.02, Fig. 2) when excluding SAPPHIRE trials [18]. This trial specifically added an embolic protection device to carotid artery stenting, which was more efficient in preventing major vascular events. Similarly, no evidence was found to show that CE protected against 1-year/within 1-year major vascular events (RR, 0.69; 95 % CI: 0.40–1.18; *P* = 0.18, Fig. 2). Substantial heterogeneity was observed in the magnitude of the effect across the trials included, according to a sensitivity analysis. It was concluded that CE was associated with a reduction in the risk of 1-year/within 1-year major vascular events, which was decreased by 44 % (RR, 0.56; 95 % CI: 0.42–0.75; *P* < 0.001, Fig. 2) when excluding SAPPHIRE trials [27]. Finally, the pooled analysis showed no significant differences in the influence of CE and CA on long-term (more than 1-year) major vascular events (RR, 1.00; 95 % CI: 0.87–1.14; *P* = 0.95, Fig. 2).

**Fig. 3:**
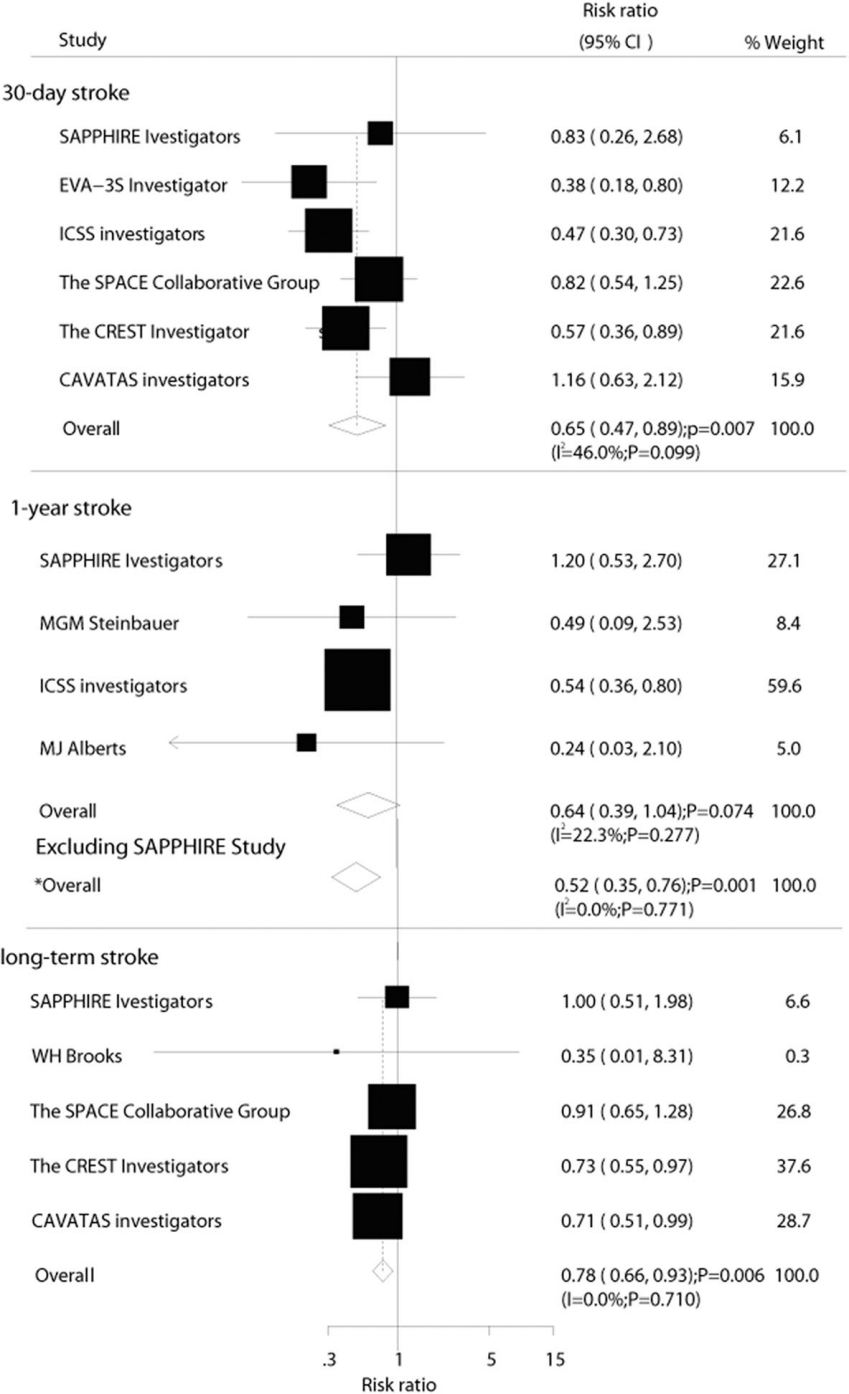
Effect of carotid endarterectomy on the risk of stroke compared with carotid angioplasty

Data on the effect of CE on 30-day stroke were available from 6 trials, including 6692 patients and 330 events of stroke. Overall, CE resulted in a 35 % reduction in the risk of 30-day stroke compared with CA (RR, 0.65; 95 % CI: 0.47–0.89; *P* = 0.007, Fig. 3). Despite some evidence of heterogeneity across the studies included, a sensitivity analysis indicated that the results were not affected by sequential exclusion of any particular trial from the pooled analysis. Furthermore, although CE reduced the risk of 1-year/within 1-year stroke by 36 %, it was not associated with a statistically significant difference (RR, 0.64; 95 % CI: 0.39–1.04; *P* = 0.07, Fig. 3). A sensitivity analysis indicated that CE was associated with a reduction in the risk of 1-year/within 1-year stroke, which was decreased by 48 % (RR, 0.52; 95 % CI: 0.35–0.76; *P* = 0.001, Fig. 3) when excluding SAPPHIRE trials [27]. Finally, when patients received CE, the risk of long-term stroke was significantly reduced by 22 % compared with CA (RR, 0.78; 95 % CI: 0.66–0.93; *P* = 0.006, Fig. 3).

**Fig. 4:**
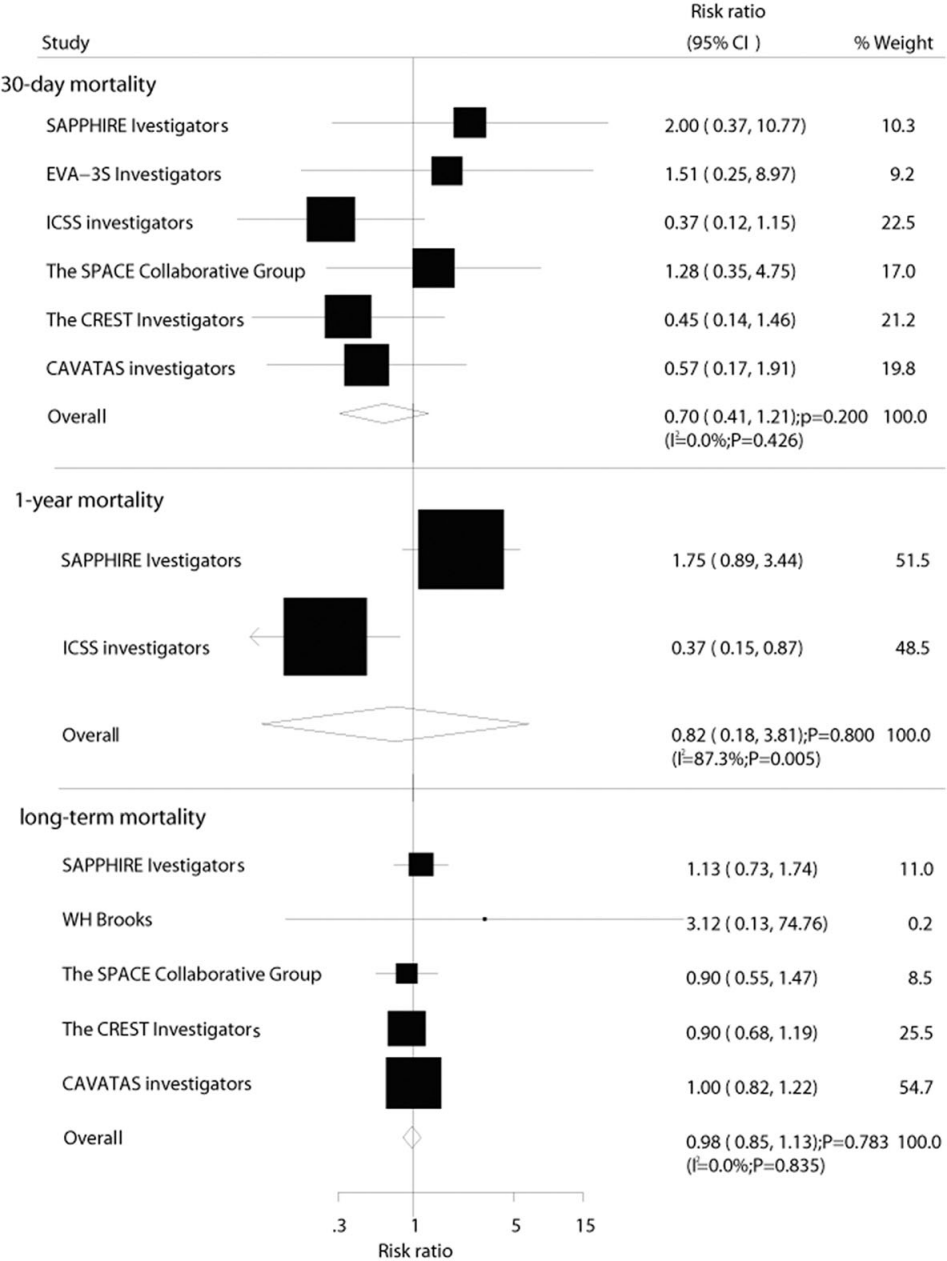
Effect of carotid endarterectomy on the risk of mortality compared with carotid angioplasty

Data for the effect of CE on 30-day mortality were available from 6 trials, which included 6692 patients and reported 59 events of death. No effect of CE on the risk of 30-day mortality was observed (RR, 0.70; 95 % CI: 0.41–1.21; *P* = 0.20, without evidence of heterogeneity, Fig. 4). Similarly, no evidence was found to show that CE could reduce the risk of 1-year/within 1-year mortality (RR, 0.82; 95 % CI: 0.18–3.81; *P* = 0.80, Fig. 4) and long-term mortality (RR, 0.98; 95 % CI: 0.85–1.13; *P* = 0.78, without evidence of heterogeneity, Fig. 4).

**Fig. 5:**
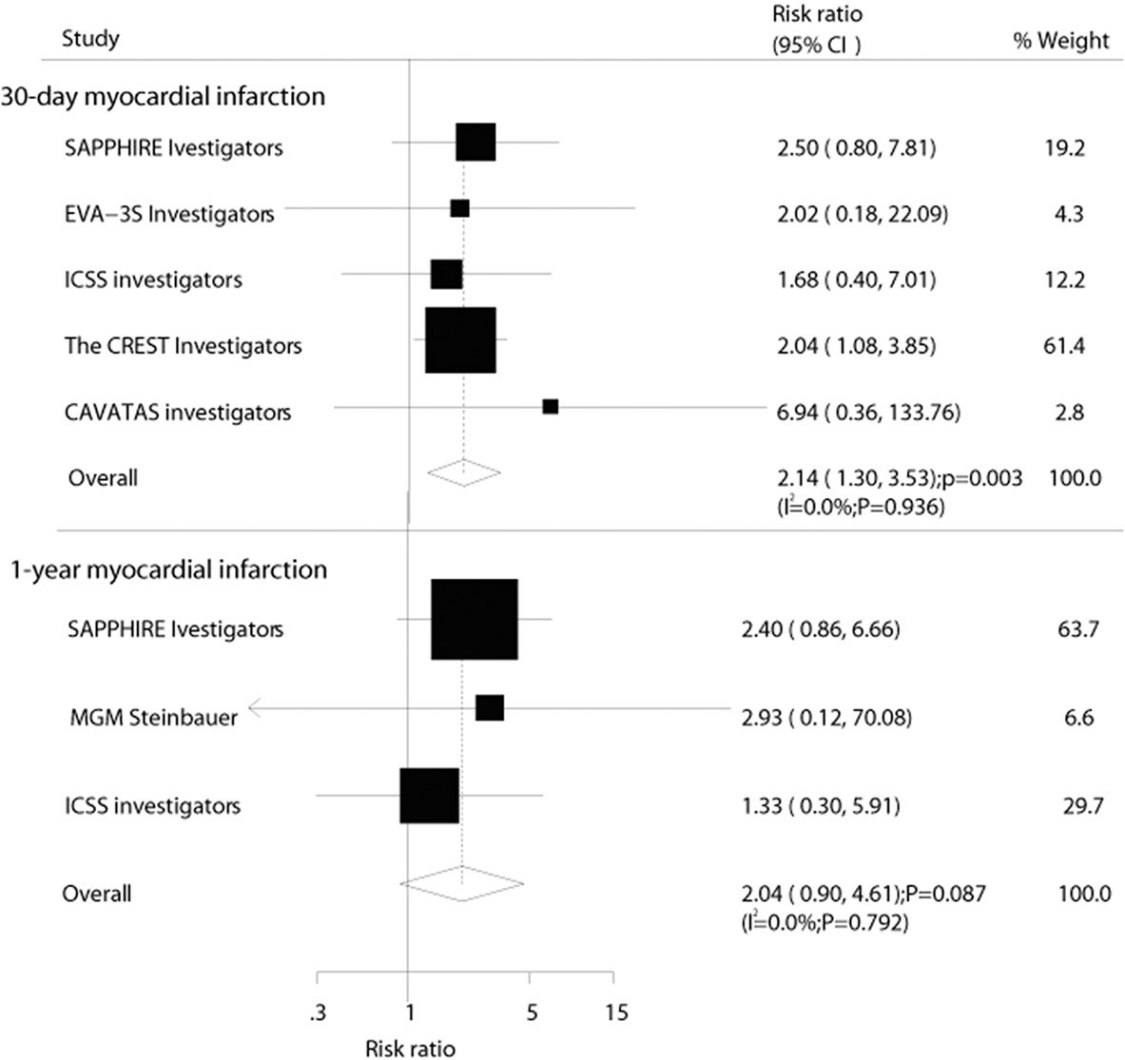
Effect of carotid endarterectomy on the risk of myocardial infarction compared with carotid angioplasty

The effect of CE on the risk of 30-day myocardial infarction was reported in 5 trials, which included 5509 patients and recorded 70 events of myocardial infarction. Overall, it was noted that CE increased the risk of 30-day myocardial infarction by 114 % compared with CA (RR, 2.14; 95 % CI: 1.30–3.53; *P* = 0.003; Fig. 5). Only three trials provided data on 1-year/within 1-year myocardial infarction. It was noted that CE increased the risk of 1-year/within 1-year myocardial infarction by 104 %, but it was not associated with a statistically significant difference (RR, 2.04; 95 % CI: 0.90–4.61; *P* = 0.09, Fig. 5). Furthermore, only SAPPHIRE trials [27] provided data on long-term myocardial infarction. No effect of CE on the risk of long-term myocardial infarction events was observed (RR, 1.56; 95 % CI: 0.69–3.49; *P* = 0.28).

Egger test [16] was used to check for potential publication bias, which showed no evidence of publication bias for the outcomes of 30-day major vascular events (*P* value for Egger test, 0.889), 30-day stroke (*P* value for Egger test, 0.902), and 30-day myocardial infarction (*P* value for Egger test, 0.376). However, evidence was found of publication bias for 30-day mortality (*P* value for Egger test, 0.025). The conclusions were not changed after adjustment for publication bias using the trim-and-fill method [28].

## Discussion

A direct relationship was observed between the degree of carotid artery stenosis and the risk of stroke. Although CE has been considered the gold standard for the treatment of carotid stenosis for decades, evidence from large-scale randomized controlled trials [6] has shown that CA has emerged as an alternative therapy for this common disorder. The results of this meta-analysis showed that CE reduced the risk of 30-day stroke and long-term stroke. However, it also significantly increased the risk of myocardial infarction compared with CA. Furthermore, sensitivity analyses suggested that CE might influence the risk of 30-day and 1-year/within 1-year major vascular events.

According to the SAPPHIRE trials [18, 27], the study suggested that CA was not inferior to CE in preventing the risk of stroke, whether short term or long term. The present results were inconsistent with large-scale randomized, controlled trials, probably because this trial specifically added an embolic protection device to carotid artery stenting, which might have contributed more efficacy in preventing the risk of stroke. Furthermore, EVA-3S trials [21, 29] indicated that in patients with symptomatic carotid stenosis of 60 % or more, the rates of death and stroke at 1 and 6 months were lower with CE than with CA. SPACE trials [23, 30] failed to prove the noninferiority of CA compared with CE in terms of the periprocedural complication rate. Furthermore, it suggested a similar effect on the risk of ipsilateral ischemic strokes between CE and CA. The present study defined that benefits could be achieved when patients with carotid artery stenosis underwent CE. However, it was also noted that CE significantly increased the risk of short-term myocardial infarction. The risk of 1-year/within 1-year myocardial infarction and long-term myocardial infarction was not observed, probably because less number of trial provided data for this result.

CE might play an important role in mortality, although a significant difference was not observed. The reason for this could be that CE also significantly increased the risk of myocardial infarction, translating into an increased risk of life-threatening events. CAVATAS trial [26, 31] indicated that more patients had stroke during long-term follow-up in the endovascular group than in the surgical group. However, the rate of ipsilateral non-perioperative stroke was low in both the groups, with no differences in the stroke outcome measures. This conclusion was in accordance with the findings of the present meta-analysis. This study was promising because randomized controlled trials were restricted to meet the inclusion criteria, and the aim was to provide the best evidence for a causal relationship.

A previous meta-analysis [32] illustrated that carotid artery stenting was inferior to CE with regard to the incidence of stroke or death for periprocedural outcomes, especially in symptomatic patients. Furthermore, it also suggested that carotid artery stenting was associated with a lower incidence of myocardial infarction. The present study also confirmed that patients who underwent CE had a high risk of myocardial infarction. However, it also indicated that CE was associated with a statistically significant reduction in the risk in stroke.

The major limitation of this study was the inherent assumptions made for the meta-analysis. The analysis used pooled data, whether from published papers or provided by individual authors. Individual patient data and original data were not available, which restricted performing more detailed relevant analysis and obtaining more comprehensive results. Additionally, no sufficient data were available on detailed effects of CE on the risk of different types of stroke. Furthermore, during the planning stages, the intention was to perform subgroup analyses on the basis of other confounders, which might affect the treatment effect. However, the results of subgroup analyses on the basis of follow-up duration might be unreliable because of smaller cohorts included. This study attempted to provide a comprehensive review on the comparison between the efficacy of CE and CA.

## Conclusions

In conclusion, CE could reduce the risk of stroke (whether short term or long term), and might influence the risk of major vascular events compared with CA. However, it also increased the risk of 30-day myocardial infarction. The present study might guide appropriate judgments about treatment approach. It also provided evidence to justify general guidelines for patients with carotid artery stenosis. It is suggested that the following factors be improved in future research: (1) the adverse effect events of clinical trials should be recorded and reported normatively and (2) myocardial infarction events should be taken into consideration for patients who underwent CE.

## Abbreviations

CA: Carotid angioplasty
CE: Carotid endarterectomy
CIs: Confidence intervals
RR: Relative risk
